# Hydraulic Safety Mechanisms Override Traditional Wood Economics in Hyper-Arid Environments

**DOI:** 10.3390/plants14172709

**Published:** 2025-09-01

**Authors:** Rodrigo S. Rios, Bárbara Silva Rojas, Danny E. Carvajal, Andrea P. Loayza

**Affiliations:** 1 Laboratorio de Ecología del Desierto, Departamento de Biología, Universidad de La Serena, La Serena 1720170, Chile; barbara.silva@userena.cl (B.S.R.); dcarvajal@ieb-chile.cl (D.E.C.); 2 Instituto de Ecología y Biodiversidad (IEB), Santiago 7770062, Chile; aloayza@ieb-chile.cl

**Keywords:** coastal desert, hyper-aridity gradient, wood trait space, wood density, hydraulic efficiency, hydraulic transport, embolism

## Abstract

Environmental stress drives plant communities toward conservative ecological strategies through increased wood density (WD) within the Wood Economics Spectrum (WES). However, hyper-arid regions like the Coastal Atacama Desert (CAD) challenge this pattern, where woody plants exhibit acquisitive traits and decreased WD with increasing aridity. The underlying anatomical mechanisms remain poorly understood. This study examined how extreme aridity in the CAD shapes wood economics strategies, testing whether anatomical changes prioritize hydraulic safety over efficiency within the WES. Six shrub communities along an aridity gradient were sampled, measuring composition and abundance of 29 species across 20 plots per site. Community-weighted means of eight wood traits—including WD, vessel density/diameter, parenchyma fraction, fiber fraction, and lumen fractions—were analyzed. PCA, triangular and linear models assessed trait variation along the aridity gradient. Unexpectedly, WD increased at both gradient extremes but through different tissue compositions, with no clear shift from acquisitive to conservative strategies. Instead, vessel traits (density and diameter) were key, reflecting an independent hydraulic safety–efficiency trade-off. PC2 strongly correlated with aridity, showing reduced vessel size and increased density under greater aridity. Findings reveal that hyper-aridity disrupts traditional wood economics, with hydraulic adaptation—not WD-mediated resource use—driving community assembly. This highlights the dominance of safety–efficiency trade-offs in structuring shrub communities in extreme deserts, emphasizing hydraulic traits over conventional resource strategies.

## 1. Introduction

Understanding how plant communities adapt to extreme environmental conditions remains a central challenge in ecology, particularly as climate change intensifies aridity across global drylands [1,2]. The Wood Economics Spectrum (WES) offers a powerful framework for predicting how woody plants balance resource acquisition and conservation strategies, primarily mediated by variation in wood density (WD) and its associated anatomical traits [3]. However, having been developed largely from mesic environments, the WES framework may be less predictive in the world’s most extreme ecosystems, where trait–environment relationships may break down under extreme abiotic stress [4]. Hyper-arid regions, such as the Atacama Desert, serve as natural laboratories for examining these limits, where shrub communities persist under conditions that challenge conventional plant functional theory and call into question the universality of established ecological theories [5,6].

Environmental filtering along aridity gradients favors drought-tolerant, resource-conservative traits [6,7], with particularly strong selection in hyper-arid ecosystems where resource scarcity constrains plant strategies [4,7]. These extreme conditions may select for unconventional trait combinations that deviate from frameworks developed for moderate climates. Selective pressures can vary non-linearly, causing threshold effects where standard trait–environment relationships break down [4,8]. Understanding these dynamics in hyper-arid systems is essential for predicting community responses to increased aridity and testing trait framework generalizability across environmental extremes.

Variation in wood density (WD) reflects a fundamental trade-off between conservative and acquisitive ecological strategies, shaped by underlying anatomical investments in xylem structure and function [9,10]. High WD typically reflects conservative strategies characterized by greater tolerance to stress and increased structural resistance to breakage or decay, whereas low WD is associated with faster growth and acquisitive resource-use [11]. These contrasting strategies arise from functional trade-offs between investment in structural support and efficiency in water transport, expressed as the relative proportions and characteristics of vessels, fibers, and parenchyma cells that comprise the xylem conduction system [12].

Wood anatomy thus provides a mechanistic basis for understanding these functional trade-offs, primarily through the role of three cellular structures. First, vessels function as conduits for water and nutrient transport; their size and density influence both hydraulic efficiency and vulnerability to embolism [13]. Vessel diameter, in particular, strongly influences water conduction efficiency, as conductivity increases exponentially with vessel width [14]. Greater hydraulic efficiency can enhance transpiration and carbon fixation rates, promoting plant growth [15,16]. However, this comes with a trade-off: fewer wide vessels improve conductivity but are more susceptible to hydraulic failure due to embolism formation [17], whereas a larger number of narrower vessels provide redundancy and increase resistance to hydraulic dysfunction [18,19]. Second, fibers serve as the primary structural support tissue. A higher proportion of fibers fraction increases WD and mechanical strength, aiding in structural integrity [20]. Third, parenchyma cells, including both axial and ray, are involved in storage and metabolic functions, and facilitate lateral transport between the xylem and phloem [21]. The relative abundance of these three cell types determines WD, with fibers being the main source of variation due to their greater abundance and contribution to cell wall thickness [22].

The Coastal Atacama Desert (CAD) provides an ideal system to study wood economics under extreme aridity. Its strong aridity gradient, drives variation in plant community composition closely tied to resource acquisition strategies [4,23], challenging traditional economics spectrum predictions. For example, Carvajal et al. [4] found that, in this hyper-arid environment, plants adopt functional strategies opposite to those predicted by traditional models—exhibiting acquisitive trait combinations in aerial organs, such as decreased WD with an increased in specific leaf area with increasing aridity. This apparent contradiction suggests that, in extremely dry environments, fast resource-use strategies may confer adaptive advantages by enabling plants to capitalize on brief windows of high resource availability, thereby increasing their survival probability [24,25].

The mechanistic basis for these inverse patterns remains unclear, particularly regarding micro-anatomical responses to aridity. Melián et al. [26] found that wood density (WD) in Argentine Monte species is driven by fiber tissue variation, reflecting trade-offs in conduction, storage, and support. However, how wood anatomy relates to ecological strategies in extreme environments is still poorly understood. Shrubs—unlike trees—exhibit multi-stemmed growth, resprouting ability, and narrower vessels, prioritizing cavitation resistance over hydraulic efficiency [27,28]. These traits may enhance survival in arid environments with unpredictable water availability.

While the WES influences plant performance via resource acquisition and stress tolerance [3,29], its role in hyper-arid communities remains unclear. Most studies focus on temperate species [30,31,32], leaving a gap in understanding community-level trait variation in extreme environments like the Atacama Desert, particularly for micro-anatomical responses to aridity. This knowledge is critical as climate change accelerates desertification [7,33,34].

This study examines how aridity-driven selective pressures shape ecological strategies associated with the WES across shrub communities in the CAD. Specifically, we examine whether increasing aridity modulates community-level wood trait spectra and their underlying trade-offs. We also identify the micro-anatomical wood traits that contribute to variation in WD and evaluate their role in functional adaptation along aridity gradients. We hypothesize that increasing aridity drives community-level shifts toward more conservative wood economics strategies, reflected in higher WD values and associated anatomical changes predicted by the WES.

In particular, we expect an increase in fiber fraction and a reduction in parenchyma content with increasing aridity, indicating greater investment in structural support and reduced allocation on storage and rapid resource transport. These anatomical changes should lead to a decrease in lumen fraction, further supporting the predicted increase in WD along the aridity gradient. At the cellular level, we predict a trade-off in vessel traits, such as size and density, shifting from traits that promote hydraulic efficiency to those that enhance hydraulic safety as aridity intensifies.

## 2. Materials and Methods

### 2.1. Study Sites and Design

This study was conducted at six sites along the CAD (30°38′ S to 26°11′ S), a hyper-arid region characterized by a pronounced aridity gradient that increases from south to north. The selected sites, each separated at least 100 km, are listed from north to south: (1) Pan de Azúcar (PA), Quebrada El León (QL), Llanos de Challe (LLA), Chañaral de Aceituno (CHA), Cuesta Porotitos (PO), and Fray Jorge (FJ). Mean annual precipitation across sites ranges from 4 to 147 mm, and mean annual temperatures fluctuate between 13.6 and 22.9 °C (Table S1). Rainfall is highly seasonal, concentrated in a few winter pulses (May–September), followed by prolonged drought periods lasting up to 10 months at wetter sites and several years at the driest ones [4].

Aridity was quantified using the “Global Aridity Index and Potential Evapotranspiration Database—Version 3” (Global-AI_PET_v3), which provides long-term (1970–2000) climate averages at a 30-arcsecond spatial resolution [35]. We used the 1–AI aridity index, calculated as 1–(annual precipitation/potential evapotranspiration), as proposed by the UNEP [36]. This index offers a simple yet robust measure of long-term climatic water deficit and is widely used to characterize site dryness [37]. Based on 1–AI, all sites were classified as hyper-arid, except for PO and FJ, which fall within the arid category for drylands (Table S1).

We measured traits from those woody shrubs species that accounted for ca. 90% of the total species abundance at each site. The sampled flora included 29 native or endemic Chilean shrub species in total, spanning eight plant orders, 13 families, and 22 genera. Species sampled per site varied between 4 and 9 (Table S2). To minimize variation unrelated to aridity, we restricted sampling to shrub communities growing on sandy soils of west facing slopes of less than 5%.

At each site, we established 20 permanent plots of 50 × 2 m, where we recorded shrub species composition, diversity and abundance. These data were used to select the species for wood trait measurements and to weight trait values by species abundance at the community level. Wood traits were quantified for all shrub species present within the plots, regardless of whether they occurred at other sites. To minimize local-scale environmental variation, plots at each site were at least 100 m apart and located in areas with similar topography, exposure, and microenvironmental conditions, allowing us to capture the effects of aridity. Moreover, at some study sites, factors such as soil water potential, nutrient limitation, and other soil properties are not aligned with our aridity gradient and do not circumscribe converge into a community-level plant economics spectrum [38].

### 2.2. Functional Trait Measurements

For all selected species at each site, a 10 cm segment of the main stem (including nodes and branching points) was collected from each of five individuals. Functional traits defining the wood functional space were estimated based on these samples. Stem segments were taken from reproductively mature and apparently healthy individuals, showing no signs of physical damage, herbivory, or pathogen attacks. Two traits measured were macro-anatomical: wood density (WD) and bark fraction (Bark). Following the standardized protocols Pérez-Harguindeguy et al. [39], WD [g·cm^3^] was estimated using the volume displacement method, while Bark was calculated as the proportion of total stem diameter made up by bark (i.e., the outer part of the stem external to the xylem, including the vascular cambium).

The remaining six traits were micro-anatomical, related to the configuration of the xylem conduction system. These were measured on 20 μm thick cross-sections obtained for all samples using a semi-automatic rotary microtome (RDW Minux^®^ Microtome S700, RWD Life Science Co., Ltd., Shenzhen, Guangdong, China). The measured traits included: vessel density (VD, vessels·μm^2^), mean vessel diameter (MVD, μm), parenchyma fraction (Parenchyma), fiber fraction (Fiber), total lumen fraction (Lumen), and lumen fraction without vessels (non-vessels). Sectioning and staining procedures followed protocols adapted from Melián et al. [26] and Yeung et al. [40] (Supplementary Materials S1).

To analyze micro-anatomical traits, we captured digital microscope images of each cross-section and superimposed three randomly placed triangular regions of interest (ROIs) radiating from the interior to the exterior. The triangular shape is ideal for cylindrical cross-sections as its apex, oriented toward the center, ensures that the ROI spans a consistent radial cell file. This procedure provided a representative transect of all tissue layers and captured the essential radial gradient for the analysis of the configuration of the xylem conduction system.

In each triangle, the three tissue types were identified and, based on staining color, the fraction area of each tissue type was quantified relative to the total area of the triangle. Therefore, fiber, parenchyma and lumen fractions were measured in percentage summing 100%. Vessels were also identified within triangles, counted (VD) and their diameter was measured as a size estimate (MVD). Analysis of tissue fraction and cell measurements were performed using ImageJ software (version 1.54m). Mean values of these traits across the three triangular sections were used as trait estimates of each individual shrub. All measured traits contribute to defining wood anatomy and plays a key role in the trade-offs between hydraulic function, storage capacity, and mechanical support (Table S3).

### 2.3. Estimation of Community-Weighted Mean Values

For each permanent plot, trait values of all species present were weighted by the log-transformed relative abundance of the corresponding species. Log transformation was applied to reduce the influence of dominant species and prevent their overrepresentation in community-weighted mean (CWM) calculations, following the approach by De Bello et al. [41]. This procedure allowed us to calculate the CWM of each trait at the plot level. Calculations were conducted using in the FD package in R [41] based on the following formula:

$$CWM=\sum_{i=1}^{N}{p_{i}x}_{i}$$

where *N* is the number of species found in a given plot, *p_i_* is the log(*p* + 1) transformed abundance of species *i* (rescaled to sum to 1 across all species in the plot), and *x_i_* is the trait value of species *i*. As a result, for each trait, we obtained 20 CWM values per site. In this calculation log(*p* + 1) transformations were used as suggested by De Bello et al. [41].

### 2.4. Data Analyses

To assess variation in the WES across shrub communities in the CAD and its relationship to increasing aridity, we performed a principal component analysis (PCA) using the vegan-package in R [42]. The analysis was based on CWM trait values at the plot level, enabling the identification of major axes of trait variation axis along the aridity gradient, as well as underlying phenotypic trade-offs. Prior to analysis, CWM values were standardized. Traits expressed as proportions/percentages were transformed using the arcsine square root method to meet assumptions of normality as they are inherently bounded between 0 and 1 or between 0 and 100.

The PCA was conducted on the resulting matrix of trait values across all six communities. Functional structure in trait space was interpreted through the eigenvectors from the first two PCA axes, which highlight trade-offs along trait variation gradients. Trait vectors pointing in opposite directions indicate functional trade-offs, while those pointing in the same direction indicate positive trait covariation. Plot scores along these vectors were used to infer changes in ecological strategies across the aridity gradient.

The PCA axis that best represented the shift from acquisitive to conservative strategies was identified based on the direction and composition of trait loadings. Aridity was projected as a supplementary variable onto the PCA ordination using the envfit() function from the vegan package in R, with 9999 permutations to assess statistical significance (*p* < 0.05) of its correlation with the PCA axes. To further assess differences in ecological strategies among sites across wood trait space, we performed a permutational multivariate analysis of variance (perMANOVA), using site as the independent factor and a Bray–Curtis distance matrix of the CWM trait values as the response variable.

For each set of CWM values per trait, individual generalized linear models (GLMs) were fitted with aridity as the predictor using the stats base package in R [43]. Both linear (*Y_i_ = ß*_0_ *+ ß*_1_*X_i_ + e_i_*) and quadratic (*Y_i_ = ß*_0_ *+ ß*_1_*X_i_ + ß*_2_*X*^2^*_i_ + e_i_*) models were evaluated to assess the shape of the relationship between CWM values and the 1–AI index. The best-fitting model for each trait was selected using the corrected Akaike Information Criterion (AICc), implemented via the model.sel() function from the MuMIn package in R [44]. The significance of individual model parameters was assessed using likelihood ratio tests (LRTs). When both linear and quadratic models had substantial support (ΔAICc < 2), we performed model averaging using the model.avg() function.

To integrate variation in lumen, fiber, and parenchyma tissue fractions across sites, we constructed a triangular model using a ternary diagram. This diagram, based on CWM values at the plot level, was generated using the R package ggtern ver. 3.5.0 [45], where axes in the triangular tri-plot represent the percentages of each tissue fraction. To evaluate what combination of CWM values of tissue fractions best explains changes in WD, we performed a multiple regression analysis based using a GLM. In this model, CWM values of lumen, fiber and parenchyma fractions were included as predictors of CWM WD, following the same GLM procedures described previously. Finally, to assess how wood trait space varies with aridity, we regressed the 1–AI index against the scores of the first two principal components (PC1 and PC2) using GLMs with both linear and quadratic terms. All statistical analyses were conducted in R version 4.3.2 [46] (Supplementary Materials S2).

## 3. Results

### 3.1. WES and Wood Trait Covariation Across Sites

**Figure 1:**
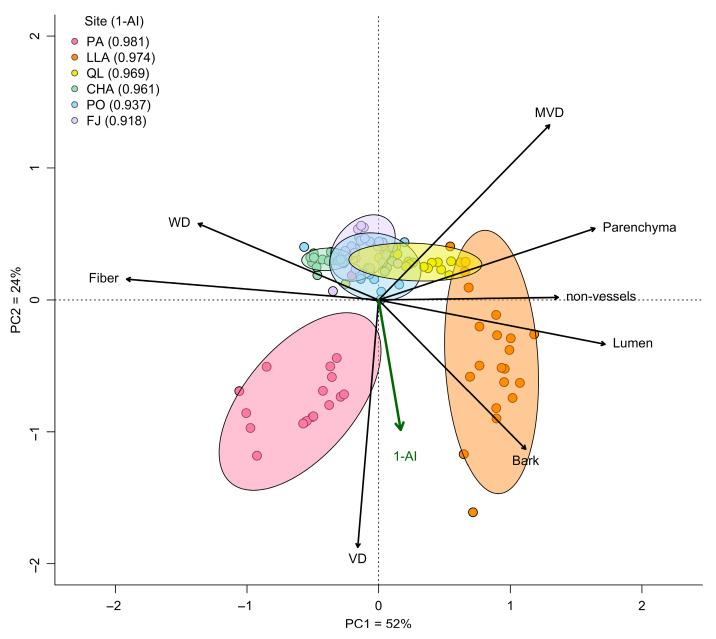
Wood Economics Space for shrub communities of the Coastal Atacama Desert, based on a principal component analysis (PCA) of eight macro- and micro-anatomical traits. The PCA was conducted on community-weighted mean (CMW) trait values from 20 plots per site across six sites. Axes represent independent functional gradients: PC1 (52%) reflects an acquisitive-conservative resource allocation spectrum, and PC2 (24%) captures a hydraulic safety–transport efficiency trade-off. Aridity, expressed as the 1–AI index, is overlaid as a green vector to show its association with wood trait space. Higher values of 1−AI indicate greater aridity. A value exceeding 0.95 denotes a hyper-arid environment, while values below this threshold are classified as merely arid for drylands. Ellipses indicate the 95% CI variation in site-level trait distributions; point colors denote individual plots within each site. Site acronyms and their corresponding 1–AI values are shown in the legend. Trait acronyms: WD, wood density; Bark, fraction of bark thickness; Fiber, fiber fraction; Lumen, total lumen fraction; Parenchyma, parenchyma fraction; non-vessels, lumen fraction excluding vessels; MVD, mean vessel diameter; VD, vessel density. For full site names and details, see the Study Sites and Design section.

Principal component analysis revealed that wood trait variation was primarily captured by the first component (PC1), which explained 52% of the total variance. PC1 represents the gradient associated with WD and all tissue fractions. Along PC1, WD was negatively correlated with lumen, parenchyma, and non-vessels, and positively correlated with fiber. The second component (PC2), accounting for 24% of the variation, was defined independently by cellular-level anatomical traits, showing a negative correlation between VD and MVD (Figure 1). Variation in aridity, measured by changes in the 1–AI index, was correlated with the multivariate trait space (envfit: R^2^ = 0.40, *p* < 0.001), particularly along PC2 (green vector in Figure 1). This axis showed a positive correlation with VD and a negative correlation with MVD, suggesting a functional adjustment of vessel traits along the aridity gradient. Bark traits contributed to both PC1 and PC2, but exhibited a trade-off with WD.

At the site level, shrub communities differed significantly in their position within wood trait space across the CAD (perMANOVA: Pseudo-F = 90.03, *p* < 0.001, R^2^ = 0.798). However, trait variation among communities did not follow a consistent shift from acquisitive to conservative strategies with increasing aridity. Only the PA community exhibited a trait combination clearly aligned with conservative resource use. In contrast, QL, CHA, PO, and FJ showed intermediate strategies, and notably, LLA—despite being the second most arid site—displayed traits consistent with an acquisitive strategy (Figure 1).

**Figure 2:**
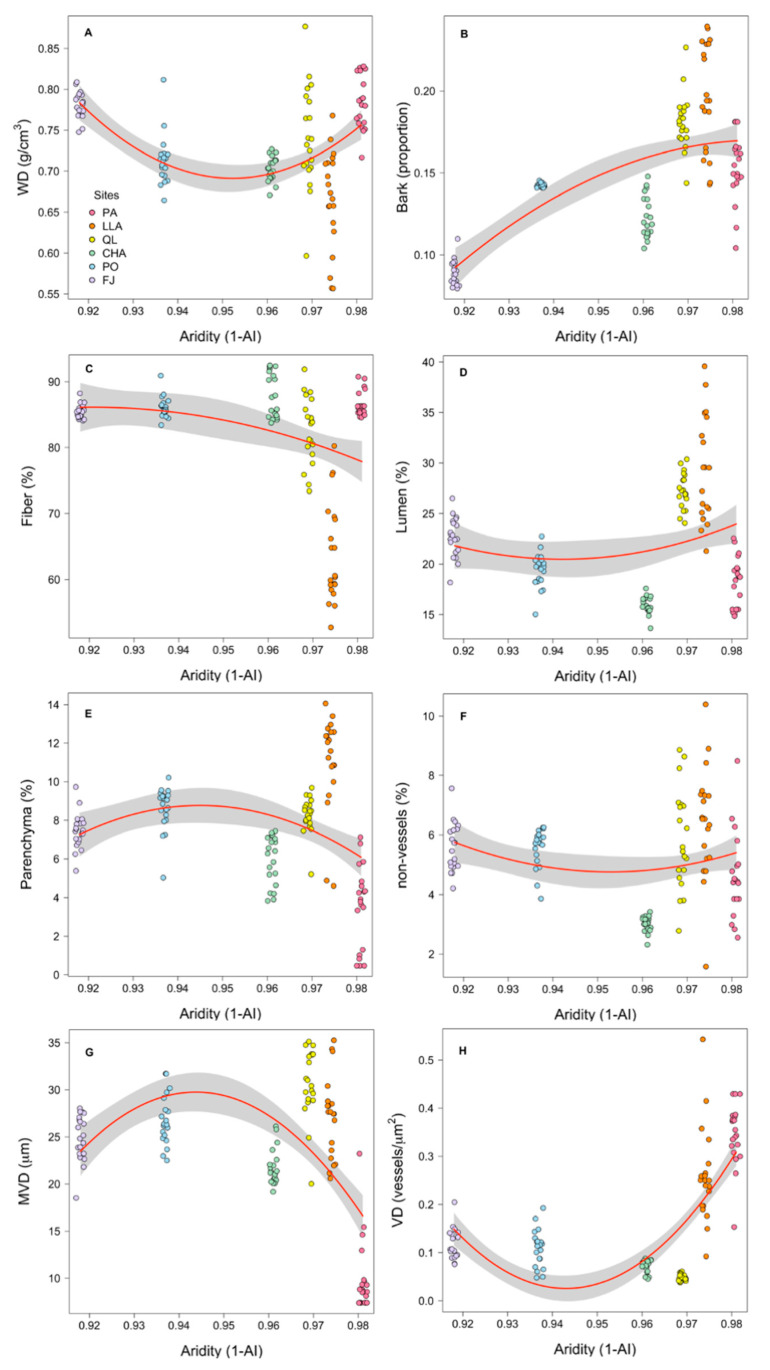
Relationships between aridity (1–AI index) and individual wood functional traits across shrub communities in the Coastal Atacama Desert. Each panel shows results from general linear models (GLMs) incorporating linear and quadratic terms. Macro-anatomical traits: (**A**) WD, wood density; (**B**) Bark, fraction of bark thickness. Tissue fraction traits are: (**C**) fiber, fiber fraction; (**D**) lumen, total lumen fraction; (**E**) parenchyma, parenchyma fraction; (**F**) non-vessels, lumen fraction excluding vessels. Hydraulic system traits: (**G**) MVD, mean vessel diameter; (**H**) VD, vessel density. Solid red lines represent significantly fitted GLMs with 95% CI intervals (gray bands). Points represent community-weighted mean values for individual plots, color-coded by site. See the Study Sites and Design section for site acronyms and Table 1 for full model statistics.

All traits exhibited variation in relation to the aridity index (1–AI) according to the GLMs (Table 1), although the nature of these relationships differed among traits (Figure 2A–H). Macro-anatomical traits showed non-linear responses to aridity. WD increased at both ends of the aridity gradient, whereas bark increased with aridity but plateaued at the most arid sites (Figure 2A,B). Tissue fractions also varied non-linearly with aridity. Fiber and non-vessels showed both linear and quadratic components (Table 1). Fiber and non-vessels slightly decreasing non-linearly with increasing 1–AI, while lumen increased non-linearly at the most arid sites (Figure 2C,D,F).

In contrast, parenchyma exhibited only a quadratic response (Table 1), declining at both ends of the aridity gradient (Figure 2E). Hydraulic traits at the cellular level exhibited the strongest model fits (MVD: R^2^ = 0.31; VD: R^2^ = 0.55; Table 1), with marked non-linear relationships with aridity (Table 1). MVD declined at both ends of the gradient, particularly under extreme aridity (Figure 2G), whereas VD increased toward the extremes, especially under the highest aridity conditions (Figure 2H).

### 3.2. Tissue Fractions and Wood Density

**Figure 3:**
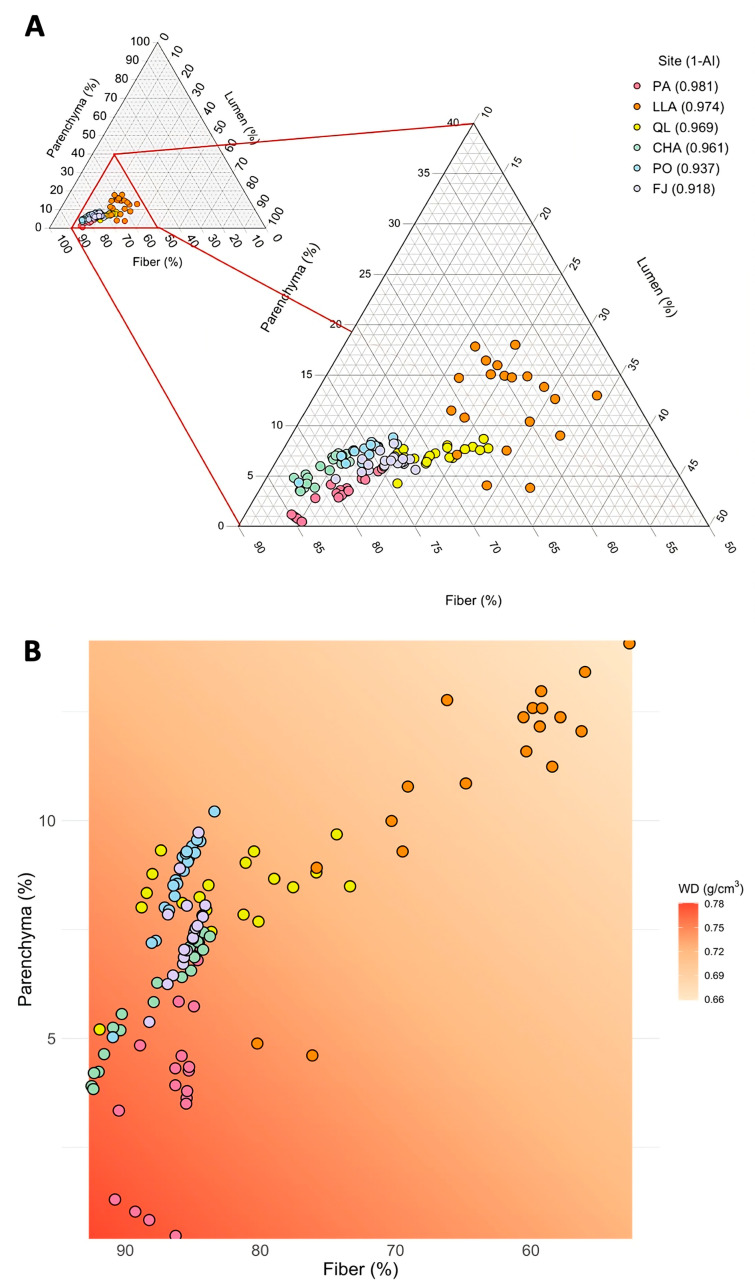
Tissue composition trade-offs and their relationship with wood density (WD) across shrub communities in the Coastal Atacama Desert. (**A**) Ternary diagram showing the distribution of community-weighted mean values of lumen, fiber, and parenchyma tissue fraction across sites. The full trait space is shown in the inset, with a zoom-in on the area where all observed values occur. (**B**) Bivariate trait space illustrating the trade-off between fiber and parenchyma fractions, overlaid with a color gradient representing WD (g·cm^−3^) fitted from a multiple regression model. Higher WD values are associated with increased fiber and decreased parenchyma fractions. Points represent plots, colored by site. See the Study Sites and Design section for site acronyms.

Tissue fraction variation across and within sites was predominantly skewed toward a single vertex of the ternary model, characterized by low parenchyma (<20%) and lumen (<35%), but high fiber (>50%)—a pattern commonly observed in desert shrubs (Figure 3A). Among plots, fiber exhibited the widest range (53–85%), followed by lumen (12–34%), and parenchyma (0.4–18%). Across sites, the combined distribution of these fractions aligned along the vertex, revealing a trade-off primarily between fiber and parenchyma. In particular, fiber content increased as parenchyma decreased, reflecting a shift towards a conservative resource-use strategy. However, these shifts were not clearly associated with aridity gradients among sites (see zoomed triangle in Figure 3A).

Variation in WD was explained by changes in tissue fractions (Figure 3B). Multiple regression model selection revealed that WD was influenced by the trade-off between fiber (χ^2^ = 0.0810, d.f. = 116, *p* < 0.001) and parenchyma (χ^2^ = 0.0211, d.f. = 116, *p* = 0.035), whereas lumen had no significant effect (χ^2^ = 0.0019, d.f. = 116, *p* = 0.40). Specifically, WD decreased with increasing fiber content (β = −0.002, SE = 0.001) and increased as parenchyma declined (β = −0.005, SE = 0.003).

### 3.3. Aridity and Wood Trait Gradients

**Figure 4:**
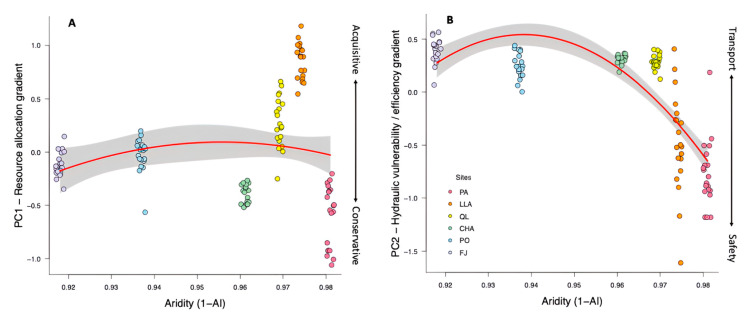
Relationships between aridity (1-AI index) and two principal functional gradients of the Wood Economics Space (WES) at the community level for shrub communities of the Coastal Atacama Desert (based on the PCA of Figure 1). (**A**) PC1 represents the acquisitive-conservative resource allocation gradient. Negative PC1 scores indicate a conservative strategy, characterized by high wood density and fiber fraction, and low parenchyma, lumen, and non-vessels tissue fractions. Positive PC1 scores represent an acquisitive strategy with opposite trait values. (**B**) PC2 represents the hydraulic safety–efficiency gradient. Negative PC2 scores correspond to a strategy favoring hydraulic safety, with high vessel density and low mean vessel diameter. Positive PC2 scores indicate a transport efficiency strategy, low vessel density and larger vessels. Solid red lines indicate significant GLMs (including linear and quadratic terms), with shaded gray bands representing 95% CI. Points represent plots, colored by site. See the Study Sites and Design section for site acronyms and Table 1 for full model statistics.

In the wood trait space, PC1 and PC2 represented two orthogonal yet interconnected gradients. PC1 captured a resource allocation gradient, reflecting trade-offs between WD and tissue fraction composition (Figure 1). This gradient varied independently of aridity, as neither linear nor quadratic relationships with 1–AI were significant (Table 1). Communities positioned at the extremes of PC1 exhibited either acquisitive and conservative strategies (Figure 4A), but these were not consistently associated with the aridity gradient. In contrast, PC2 represented a hydraulic vulnerability–efficiency gradient, defined by the trade-off between MVD and VD (Figure 1).

Unlike PC1, the hydraulic gradient exhibited a significant non-linear relationship with aridity (Table 1). Along PC2, communities transitioned from high MVD (reflecting hydraulic safety) to high VD (reflecting hydraulic efficiency), as indicated by shifts from positive to negative PC2 scores. This suggests that under increasing aridity, shrubs tend to prioritize hydraulic safety over efficiency, particularly at the most arid end of the gradient (Figure 4B).

## 4. Discussion

Our study shows that shrub communities across the CAD do not exhibit the expected shift from acquisitive to conservative wood economics strategies along the aridity gradient when considering resource allocation traits, such as wood density and tissue fraction composition. This challenges conventional WES predictions [3]. Instead, we found complex, non-linear wood trait patterns, suggesting that resource-use strategies are context-dependent and are shaped by local species composition and specific adaptations, rather than by climatic factors alone, under extreme environmental conditions.

In contrast, shrub communities exhibited consistent adjustments to aridity along a self-regulating phenotypic gradient shaped by the expected trade-off between hydraulic efficiency and vulnerability. This adaptative gradient arises from a cellular-level trait trade-off (MVD vs. VD), with communities shifting from transport efficiency to hydraulic safety under increasing aridity. These results suggest that shrub communities can adjust resource allocation across tissues in idiosyncratic ways provided that hydraulic safety is maintained as aridity increases.

**Table 1:** Results from adjusted general linear models (GLMs) evaluating the relationship between aridity (1–AI index) and wood functional traits across shrub communities in the Coastal Atacama Desert. For each trait, both linear and quadratic models were fitted, and model performance was compared using log-likelihood (logLik), corrected Akaike Information Criterion (AICc), difference in AICc relative to the best model (ΔAICc), and Akaike weights (*w_i_*). Pseudo-R^2^ values indicate model fit. Trait abbreviations: WD, wood density; bark, fraction of bark thickness; fiber, fiber fraction; lumen, total lumen fraction; parenchyma, parenchyma fraction; non-vessels, lumen fraction without the vessels; MVD, mean vessel diameter; VD, vessel density; PC1, Acquisitive–conservative resource allocation gradient; PC2, Hydraulic safety–efficiency gradient.

Variable	Model	Slope Parameter ^1^		Pseudo-R^2^	d.f.	logLik	AICc	ΔAICc	*w_i_*
		1-AI	(1-AI) ^2^						
WD	Linear	**−0.48**	-	0.03	3	172.73	−339.30	30.16	0.00
	Quadratic	**−150.00**	**78.78**	0.26	4	188.88	−369.40	0.00	1.00
Bark	Linear	1.24	-	0.48	3	255.85	−505.50	3.18	0.17
	Quadratic	**33.30**	**−16.89**	0.50	4	258.51	−508.70	0.00	0.83
Fiber	Linear	**−129.70**	-	0.10	3	−430.61	867.40	0.00	0.64
	Quadratic	**4178.00**	−2269.00	0.11	4	−430.10	867.50	1.13	0.36
	^2^ Average	1432.47	−822.85	-	-	-	-	-	-
Lumen	Linear	32.57	-	0.02	3	−372.53	751.30	0.63	0.42
	Quadratic	−4344.00	**2305.00**	0.04	4	−371.14	750.60	0.00	0.58
	^2^ Average	−2498.73	1333.35	-	-	-	-	-	-
Parenchyma	Linear	−17.56	-	0.02	3	−292.65	591.50	6.60	0.04
	Quadratic	**3925.00**	**−2077.00**	0.09	4	−288.28	584.90	0.00	0.96
non-vessels	Linear	−6.02	-	0.01	3	−229.60	465.40	1.68	0.30
	Quadratic	−1563.00	**820.40**	0.04	4	−227.69	463.70	0.00	0.70
	^2^ Average	−1093.57	572.87	-	-	-	-	-	-
MVD	Linear	**−103.40**	-	0.10	3	−403.37	812.90	29.36	0.00
	Quadratic	**17,890.00**	**−9476.00**	0.31	4	−387.62	783.60	0.00	1.00
VD	Linear	**2.42**	-	0.21	3	99.48	−192.80	65.89	0.00
	Quadratic	**−370.40**	**196.40**	0.55	4	133.50	−258.60	0.00	1.00
PC1	Linear	2.45	-	0.01	3	−88.09	182.40	0.00	0.50
	Quadratic	362.00	−189.40	0.03	4	−87.02	182.40	0.00	0.50
	^2^ Average	182.08	−94.62	-	-	-	-	-	-
PC2	Linear	**−14.32**	-	0.39	3	−58.95	124.10	47.78	0.00
	Quadratic	**1224.00**	**−652.20**	0.60	4	−33.99	76.30	0.00	1.00

^1^ Significant slope estimates based on likelihood ratio tests (LRT, *p* < 0.05) are shown in bold. ^2^ Averaged parameter estimates are reported when both linear and quadratic models had substantial support (ΔAICc < 2). See Figure 2 and Figure 4 for graphical representation of these relationships.

### 4.1. Implications for the Wood Economics Spectrum

Our PCA of wood trait variation revealed two orthogonal axes defining the wood economics space of CAD shrub communities. The primary axis (PC1), which explained 52% of the variation, represented a resource allocation gradient characterized by trade-offs between WD and tissue fractions. Notably, this gradient varied independently of aridity, reinforcing emerging evidence that above-ground traits in hyper-arid ecosystems may deviate from theoretical expectations. While Carvajal et al. [4] reported a shift from conservative to acquisitive strategies—reflected by decreasing WD—with increasing aridity, our expanded sampling, which included more extreme sites, revealed a non-linear pattern. Specifically, WD initially decreased as previously documented [4], but increased again at the most arid sites, suggesting a return to conservative strategies under extreme conditions.

The non-linear response underscores the context-dependence of resource-use strategies across communities. For instance, the PA community (most arid) exhibited traits consistent with a conservative strategy, while LLA (second most arid) showed an acquisitive strategy. Intermediate communities (QL, CHA, PO, FJ) displayed diverse strategies not clearly aligned with their positions along the aridity gradient. These outcomes challenge Reich’s [6] predictions of consistent trait-aridity relationships and instead support Chave et al.’s [3] contention that WD often shows weak, variable or context-dependent correlations with environmental gradients.

Idiosyncratic patterns of resource allocation along aridity gradients may be a common feature of hyper-arid woody plant communities. Previous studies have documented divergent resource-use strategies between above- and below-ground traits within individual species or localities [4,47], suggesting that while hyper-aridity may drive above-ground trait selection, below-ground resource acquisition is likely shaped by different selective pressures [38]. This decoupling implies that wood trait allocation depends not only on climatic factors but also on site-specific conditions and the particular adaptive strategies of constituent species.

Within the CAD, shrub species with contrasting above-ground adaptations to water scarcity often co-occur. For instance, at LLA—acquisitive communities dominated by low WD—include stem- and leaf-succulent species such as *Oxalis gigantea*, which maintains above-ground tissues year-round, alongside drought-deciduous species like *Encelia canescens* and *Polyachyrus fuscus*, which shed all above-ground biomass during dry periods.

Despite their distinct ecological strategies, these species share similarly low to intermediate WD values. Likewise, at PA, conservative communities with higher WD, include leaf-succulent species such as *Tetragonia angustifolia* and more perennial shrubs like *Gypothamnium pinifolium* and *Heliotropium pycnophyllum*; both functional types are characterized by high WD. This functional diversity highlights the multiplicity of strategies available to cope with aridity. Consequently, community-level resource allocation strategies may arise independently of aridity, with either conservative or acquisitive syndromes prevailing based on local species composition rather than climatic filtering alone.

The multidimensional nature of wood trait variation reveals that the WES encompasses a diversity of adaptive solutions to environmental gradients, shaped by tissue-specific patterns of resource allocation. Notably, key trait relationships within the functional space—particularly those forming axes orthogonal to the primary resource allocation gradient—reveal how communities may respond to hyper-aridity. The second principal component (PC2), which explains 24% of variation and correlates significantly with aridity, captures this response through cellular-level trade-offs in vessel characteristics. Previous studies have shown that vessel traits can be independent of wood density in shrub species [48,49], and our results reinforce this decoupling. Specifically, the hydraulic vulnerability-efficiency gradient (reflected in PC2) defined by the trade-off between VD and MVD, indicates a shift from hydraulic efficiency to safety with increasing aridity, independent of the resource allocation axis.

Safety may be, in addition, reinforced by bark thickness, which increases with aridity, providing mechanical strength and insulation against desiccation [50], assuming some of the roles of WD. These patterns confirm earlier reports of weak integration between WD and vessel traits in shrub communities [12]. The independence between PC1 and PC2 suggests that shrub species can maintain self-regulating resource allocation strategies along aridity gradients, as long as hydraulics is preserved. This functional decoupling pinpoints the capacity of shrubs to balance diverse resource-use strategies with essential hydraulic constraints, a mechanism likely crucial for persistence under highly variable and stressful environmental conditions.

### 4.2. Anatomical Mechanisms Underlying Non-Linear Responses

The observed non-linear patterns in WD across the aridity gradient reflect distinct anatomical adjustments that serve different functional priorities. Although WD increased at both ends of the gradient, the underlying tissue configurations differed markedly. At the hyper-arid PA site, high WD was associated with high fiber fraction and reduced parenchyma, indicative of a classic drought tolerance strategy that enhances mechanical support and resistance to xylem cavitation. In contrast, at the more mesic FJ site, comparable WD values were achieved through increased parenchyma allocation, suggesting a metabolically active strategy that prioritizes water storage and tissue maintenance along with structural requirements.

The above results underscore that WD is an emergent property arising from multiple combinations of tissue fractions, rather than a unidimensional trait. Moreover, they support the premise by Ziemińska et al. [22], who showed that species with intermediate WD can arrive to similar WD values via different anatomical routes. The pronounced tissue-level variation we observed across sites likely reflects a broad spectrum of ecological strategies [20], challenging the conventional interpretation of WD as a consistent indicator of plant economic strategies.

Wood density in shrub communities showed a stronger association with fiber fraction than with vessel traits, confirming that its anatomical basis is primarily determined by structural support tissues rather than by conductive elements [48]. In contrast, vessel characteristics exhibited pronounced and diverse responses to aridity: MVD decreased at both ends of the gradient extremes, while VD increased under the most arid conditions. This suggests that intermediate aridity levels allow for hydraulic optimization via larger vessels, whereas extreme environments drive distinct adaptive responses. At the hyper-arid PA site, reduced VD likely minimizes embolism risk by favoring safer xylem configurations, whereas at the mesic FJ site, narrower vessels may help regulate water transport under fluctuating availability.

Parenchyma fraction displayed a unimodal relationship with aridity, reflecting its dual role in water and carbohydrate storage. Maximum parenchyma investment occurred at intermediate aridity levels, where it likely provides critical reserves to buffer environmental variability. This investment becomes constrained at both gradient extremes—limited in hyper-arid conditions by the need to reduce metabolic costs, and in less arid sites where alternative structural or physiological strategies may be more advantageous.

### 4.3. Community-Level Functional Strategies

The triangular model of tissue fractions revealed that desert shrub communities are predominantly characterized by low parenchyma (<20%) and lumen (<35%), but high fiber content (>50%)—a pattern typical of structurally robust shrub species in arid environments [26]. However, the primary trade-off occurred between fiber and parenchyma, rather than following a predictable aridity-driven pattern involving lumen fraction [51,52]. This further supports the observed decoupling between the WD allocation gradient and trait vessels. Such independence suggests that environmental filtering operates through general constraints on functional trait combinations, rather than selecting for a single, directional strategy along the aridity gradient.

The lack of a direct association between the resource allocation gradient (PC1) and aridity indicates that multiple functional solutions can persist under similar environmental conditions, provided that critical physiological requirements—such as hydraulic safety—are met through alternative trait combinations. This highlights the role of functional redundancy in resource allocation as a potential mechanism promoting community stability in hyper-arid and environmentally variable systems. Coexistence among species with divergent resource-use strategies may be facilitated by their ability exploit different aspects of the resource environment or occupy distinct temporal niches.

### 4.4. Implications for Plant Economics Theory

Evidence suggests that the WES in hyper-arid environments (e.g., CAD) should not be interpreted as a universal axis of ecological strategies, but rather as a functional dimension that may shift non-linearly—or even become decoupled—under extreme environmental conditions. In such contexts, the traditional fast-slow continuum [6] proposed for wood traits should also be interpreted considering species-specific adaptations to water scarcity, thermal stress, and resource pulse dynamics.

Our results are consistent with emerging evidence showing that extreme environments can give rise to functional strategies that diverge from classical predictions [4]. Rather than uniformly selecting for conservative strategies, hyper-aridity appears to favor a range of solutions that optimize different aspects of resource acquisition, storage, and conservation, contingent on local environmental filters and species-specific constraints, as long as hydraulic safety is maintained. Similar patterns have been found in other arid environments, supporting our hypothesis of context–dependent trait relationships [53,54,55]. However, the absence of detailed wood anatomical studies in deserts prevents comprehensive global comparisons.

Future research should prioritize on linking these functional patterns to plant performance under varying aridity regimes. In particular, the trade-off between hydraulic efficiency and safety emerges as a central axis in shaping shrub communities along arid gradients. Understanding how this trade-off constrains key attributes such as plant height, above-ground biomass, and growth rates will be essential to refining our understanding of how woody plant communities function and persist under increasingly variable and stressful environmental conditions.

## 5. Conclusions

This study reveals three critical ecological insights that challenge conventional plant functional theory. First, extreme hyper-arid conditions disrupt traditional WES predictions, as shrub communities did not exhibit the expected shift from acquisitive to conservative strategies along aridity gradients, instead display complex non-linear patterns where wood density increases at both gradient extremes through different anatomical mechanisms. Second, hydraulic safety mechanisms override resource allocation strategies as environments become more extreme, with vessel traits (VD and MVD) emerging as the primary drivers of community assembly, reflecting an independent hydraulic safety–efficiency trade-off that operates orthogonally to traditional wood economics axes.

Third, functional decoupling enables adaptive flexibility under environmental stress, as shrub communities can maintain diverse resource-use strategies provided hydraulic constraints are preserved, suggesting that multiple anatomical pathways can achieve similar functional outcomes in hyper-desert environments, such as the CAD. Our results have profound implications for predicting plant community responses to increasing aridity under climate change, highlighting that conventional trait–environment relationships may break down in extreme ecosystems where hydraulic adaptation becomes one of the dominant selective responses.

## Data Availability

Data are contained within the article and Supplementary Materials.

## Supplementary Materials

The following supporting information can be downloaded at: https://www.mdpi.com/article/10.3390/plants14172709/s1, Table S1: description of sampling sites; Table S2: Species list; Table S3: description of measured traits; Supplementary Materials S1: Protocol; Supplementary Materials S2: R code.
