# Supplementary material for: Hydraulic Safety Mechanisms Override Traditional Wood Economics in Hyper-Arid Environments
Source: Plants (Basel). 2025 Sep 1;14(17):2709. doi: 10.3390/plants14172709 (PMC12429929; doi:10.3390/plants14172709)
Supplement: Supplementary file 1 [file plants-14-02709-s001.zip › plants-3797106-supplementary.pdf]

Supplementary material

Table S1. Sampling sites, acronyms, aridity index (1−AI), and climatic classification. List of localities sampled across the aridity gradient in the Coastal Atacama Desert. Acronyms correspond to sites codes used throughout the manuscript. The 1−AI index indicates aridity, with higher values reflecting more arid conditions. Sites are categorized based on their climatic classification

Locality	Acronym	1−AI	Classification
Pan de Azúcar	PA	0.981	Hyper arid
Quebrada El León	QL	0.969	Hyper arid
Llanos de Challe	LLA	0.974	Hyper arid
Chañaral de Aceituno	CHA	0.961	Hyper arid
Porotitos	PO	0.937	Hyper arid
Fray Jorge	FJ	0.918	Arid

Table S2. Species list with taxonomic classification, collection site, and paraffin section availability. List of species sampled for anatomical analyses, including abbreviation code, taxonomic classification (Order, Family, Genus, Species), collection site, and whether paraffin-embedded sections were obtained for each species.

Code	Order	Family	Genus	Species	Site	Paraffin cut
Ab	Fabales	Fabaceae	Adesmia	*A. bedwellii*	FJ	
Ba	Asterales	Asteraceae	Bahia	*B. ambrosioides*	PO	x
Bp	Geraniales	Francoaceae	Balbisia	*B. peduncularis*	LLA, CHA	
Bpa	Asterales	Asteraceae	Baccharis	*B. paniculata*	FJ, PO	
Ca	Malvales	Malvaceae	Cristaria	*C. aspera*	LLA, PO	x
Cd	Caryophyllales	Polygonaceae	Chorizanthe	*C. deserticola*	PO	x
Cf	Polygonales	Polygonaceae	Chorizanthe	*C. frankenoides*	CHA	x
Cg	Malvales	Malvaceae	Cristaria	*C. glaucophylla*	FJ	
Cu	Asterales	Asteraceae	Chuquirag	*C. ulcina*	QL, CHA	
Ec	Asterales	Asteraceae	Encelia	*E. canescens*	QL, LLA, CHA	
Esp	Asterales	Asteraceae	Erigeron	*E. fasciculatus*	PO	
Fr	Caryophyllales	Frankeniaceae	Frankenia	*F. chilensis*	LLA	
Gp	Asterales	Asteraceae	Gypothamnium	*G. pinifolium*	PA	
Hf	Boraginales	Boraginaceae	Heliotropium	*H. floridum*	QL, LLA	
Hp	Asterales	Asteraceae	Haplopappus	*H. parvifolius*	PO	
Hpi	Boraginales	Boraginaceae	Heliotropium	*H. pycnophyllum*	PA	x
Lyc	Solanales	Solanaceae	Lycium	*L. chilense*	FJ	x
Nd	Solanales	Solanaceae	Nolana	*N. divaricata*	CHA	
Ns	Solanales	Solanaceae	Nolana	*N. sedifolia*	CHA	

Og	Oxalidales	Oxalidaceae	Oxalis	*O. gigantea*	LLA	x
Op	Asterales	Asteraceae	Ophryosporus	*O. paradoxus*	FJ	
Ot	Asterales	Asteraceae	Ophryosporus	*O. triangularis*	LLA	
Pc	Asterales	Asteraceae	Proustia	*P. cuneifolia*	FJ	
Pf	Asterales	Asteraceae	Polyachyrus	*P. fuscus*	QL, LLA	
Pp	Asterales	Asteraceae	Polyachyrus	*P. poeppigii*	PO	
Sa	Gentianales	Apocynaceae	Skytanthus	*S. acutus*	PA, QL, LLA	
Sc	Fabales	Fabaceae	Senna	*S. cumingii*	PO	
Ta	Caryophyllales	Aizoaceae	Tetragonia	*T. angustifolia*	PA, QL	
Tm	Caryophyllales	Aizoaceae	Tetragonia	*T. maritima*	CHA	

Table S3. Anatomical and functional stem traits measured in shrub communities along the aridity gradient. Their contribution to wood economics space, as principal component scores in PC1 and PC2, is also shown.

Trait acronym	Trait	Functional role	Acquisitive value	Conservative value	Units (if applicable)	PC1	PC2
MVD	Mean Vessel Diameter	Hydraulic conductivity	High	Low	µm	-1.34	1.34
Fiber	Fiber Fraction	Mechanical support	Low	High	% (of cross-sectional area)	-1.89	0.30
Parenchyma	Parenchyma Fraction	Storage, metabolic activity	High	Low	% (of cross-sectional area)	1.62	0.59
Lumen	Total Lumen Fraction	Water conduction and storage	High	Low	% (of cross-sectional area)	1.72	-0.35
non-vessels	Lumen Fraction without the Vessels	Storage capacity	High	Low	% (of cross-sectional area)	1.32	0.04
DV	Vessel Density	Hydraulic safety	Low	High	number·μm^{-2}	-0.31	-1.85
WD	Wood Density	Hydraulic safety, hydraulic conductivity, mechanical support and storage capacity	Low	High	g·cm^{-3}	-1.37	0.47
Bark	Fraction of Bark Thickness	Protection, storage, mechanical flexibility	Low	High	% (of cross-sectional diameter)	1.12	-1.13

Supplementary S1.

Stem Sample Preparation Protocol for Anatomical Analysis and Paraffin Embedding Protocol

1. Collection and Fixation:

Stem samples should be basal and lignified. Immediately after collection, place samples in a FAA solution (formalin 25%, ethanol 50%, acetic acid 25%) in Falcon tubes. Store at room temperature for 1 to 4 weeks.

2. Laboratory Dehydration:

Transfer samples to 70% ethanol and incubate at 60 °C for three days. Replace with fresh 70% ethanol and let sit for another two days.

Reduce the concentration to 50% ethanol and incubate at 60 °C for one to two days.

Subsequently, switch to 30% ethanol at 60 °C and incubate for one day. After this, either replace the ethanol with water or proceed directly to sectioning.

3. Sample Preparation for Sectioning:

Remove the bark and trim samples to appropriate sizes. If the tissue remains too hard, immerse in a softening solution composed of 1-part 95% ethanol, 1-part distilled water, and 3-parts glycerin, and incubate in an oven at 60 °C for at least 24 hours.

During sectioning, handle samples with fine brushes and moisten regularly with softening solution. Replace the blade frequently to ensure clean, high-quality sections.

4. Sectioning with Microtome:

Begin with cuts between 90 - 50 µm to level the surface, then gradually reduce the thickness to 20 µm.

5. Staining and Dehydration:

Pass the sections through the following staining and dehydration series:

50% ethanol for 3 min

Distilled water for 5 min

Safranin for 5 min

Distilled water (two rinses, 5 min each)

70% ethanol (two immersions, 3 min each)

85% ethanol (two immersions, 3 min each)

100% ethanol (two immersions, 3 min each)

NeoClear for 3 min

6. Mounting:

Position, stained sections on microscope slides, add a drop of NeoMount, and place a coverslip at a 45° angle to avoid air bubble formation.

Allow the slides to dry for at least 12 hours before observing under a microscope.

Paraffin Embedding Protocol

1. Fixation:

Place two stem cross-sections per individual in Falcon tubes, labeled with species, site, and replicate ID. Fill tubes with FAA (25% formalin, 50% ethanol, 25% acetic acid), and fix samples for 24 to 72 hours before processing.

2. Dehydration:

After fixation, rinse samples with distilled water and transfer through an ascending ethanol series:

50% ethanol for 30 min

70% ethanol for 30 min

80% ethanol for 30 min

90% ethanol for 30 min

96% ethanol for 30 min

100% ethanol for 30 min

3. Clearing:

Transfer samples to pure Neo-Clear (used as a xylene substitute) for 20 minutes to remove ethanol and prepare for paraffin embedding.

4. Paraffin Embedding:

Embed samples in an oven at ~60 °C using melted paraffin. Keep containers open during embedding. Perform three paraffin changes of 8 hours each. Ensure fast transfers to prevent paraffin solidification.

Place embedded samples into molds labeled with sample data and fill with fresh paraffin. Adjust sample position with a heated needle. Allow paraffin to solidify at room temperature; use cold water to accelerate solidification.

5. Sectioning:

Use a rotary microtome to obtain thin sections (~20 µm). Transfer ribbons to black cardstock (opaque side up), then trim sections to appropriate dimensions for mounting.

6. Preliminary Mounting:

Heat slides to 40 °C. Add a drop of distilled water to each labeled slide. Using a wet brush, transfer tissue sections (shiny side down), anchoring one end first to minimize air bubbles. Once expanded and free of compression marks, remove excess water with a brush and paper towel. Dry slides on a 30 °C warming plate for 12 hours. Slides can be stored or stained later. Alternatively, sections can be glued with Haupt's adhesive and left to dry for 12 hours.

7. Staining:

Remove paraffin by passing slides through Neo-Clear followed by a descending ethanol series (100% to 50%). Stain sections with 0.05% safranin in water containing 2% sodium chloride, followed by fast green in absolute ethanol, clove oil, and methyl cellosolve or methyl cellulose.

8. Final Mounting:

After staining, mount sections with NeoMount and allow to dry for at least 12 hours prior to light microscopy.

Supplementary S2.

R script used in statistical data analyses

```
#
# "Hydraulic Safety Mechanisms Override Traditional Wood
#        Economics in Hyper Arid Environments"
#              Rios et al. plants 2025
#             (PCA, Ternary plot and GLMs)
#-----------------------------------------------------

rm(list=ls()) # Clean internal memory of R
setwd("insert directory")

# Load required libraries
library(ggplot2)
library(MuMIn)
library(visreg)
library(viridisLite)
library(vegan)
library(readxl)
library(Ternary)
library(easystats)

# Load your data (adjust path as needed)
stem <- read.table("data.txt", header = TRUE)

#transform proportion/percentage variables
stem$fiber<- sqrt(asin(stem$fiber/100))
stem$parenchyma<- sqrt(asin(stem$parenchyma/100))
stem$total.lumen<- sqrt(asin(stem$total.lumen/100))
stem$Lumen.NoVessels<- sqrt(asin(stem$Lumen.NoVessels/100))
stem$vessels<- sqrt(asin(stem$vessels/100))
stem$prop.bark<- sqrt(asin(stem$prop.bark/100))
str(stem)
names(stem)

# Perform the PCA
#-----------------------------------------------
PCA <- stem
pca_all <- rda(PCA, scale = T, center = F)
summary(pca_all)

# Extract PC scores
site_scores <- scores(pca_all, display = "sites")
wood$PC1 <- site_scores[, 1]
wood$PC2 <- site_scores[, 2]


#-- Biplot figure --
#-----------------------------------
PCA$Site <- as.factor(stem$Site)
Site <- with(PCA, levels(Site))
PCA$Site

# Define color scheme
library(paletteer)
col <- paletteer_c("grDevices::Set 3", 30)[1:4]
leg <- paletteer_c("grDevices::Pastel 1", 30)[5:10]

col_MVD <- "black"
col_fibre <- "black"
col_parenchyma <- "black"
col_Lumen <- "black"
```

```
col_DV <- "black"
col_WD <- "black"
col_prop.bark <- "black"
col_vessels <- "black"

# point colors
colors <- c(rep("#EE799F", 20), rep("#EEEE00", 20), rep("#FF8C00", 20),
            rep("#B1E0B7", 20), rep("#ADDBF4", 20), rep("#DACEFB", 20))

# Legend colors
colo <- c("#EE799F", "#FF8C00", "#EEEE00", "#B1E0B7", "#ADDBF4", "#DACEFB")

# Ellipse colors (in alphabetical order)
elli <- c("#B1E0B7", "#DACEFB", "#FF8C00", "#EE799F", "#ADDBF4", "#EEEE00")


#--- PCA plot (axes 1 and 2) --- ****

png("Fig01.png", width=9, height=8, units="in", res=600)
par(mfrow=c(1,1), mar=c(3,3,1,1), bty="L", mgp=c(2,0.8,0)) # mas espacio
plot(pca_all, scaling = 2, choices=c(1,2), type="n",  ylim=c(-2,2), xlim=c(-2,2), main="",
xlab="PC1 = 52%", ylab="PC2 = 24%")

# Add points
with(PCA, points(pca_all, display = "sites",  scaling = 2, pch = 21, bg = colors, cex=1.5))

# Confidence intervals per group
ordiellipse(pca_all, choices = c(1, 2),  groups = PCA$Site, draw = "polygon", col = elli,
kind = "sd", conf=0.9)

# Generate vectors
g <- scores(pca_all, display = "species", choices=1:4)
len <- 1
off_factor <-0.5 # Displace vector name
arrows(0, 0, len * g[c(1,3), 1],  len * g[c(1,3), 2], length = 0.05, col = c(col_MVD,
col_parenchyma), lty=1, lwd=2)
arrows(0, 0, len * g[c(2,4), 1],  len * g[c(2,4), 2], length = 0.05, col = c(col_fibre,
col_Lumen), lty=1, lwd=2)
arrows(0, 0, len * g[c(5,6), 1],  len * g[c(5,6), 2], length = 0.05, col = c(col_DV, col_WD),
lty=1, lwd=2)
arrows(0, 0, len * g[c(7,8), 1],  len * g[c(7,8), 2], length = 0.05, col = c(col_prop.bark,
col_vessels), lty=1, lwd=2)
text(x=g[1,1], y=g[1,2], labels="WD", col = "black", pos=2)
text(x=g[2,1], y=g[2,2], labels="Bark", col = "black", pos=1)
text(x=g[3,1], y=g[3,2], labels="MVD", col = "black", pos=3)
text(x=g[4,1], y=g[4,2], labels="VD", col = "black", pos=1)
text(x=g[5,1], y=g[5,2], labels="Fiber", col = "black", pos=2)
text(x=g[6,1], y=g[6,2], labels="Parenchyma", col = "black", pos=4)
text(x=g[7,1], y=g[7,2], labels="Lumen", col = "black", pos=4)
text(x=g[8,1], y=g[8,2], labels="non-vessels", col = "black", pos=4)

# Add legend
loc <- c("PA (0.981)", "LLA (0.974)", "QL (0.969)", "CHA (0.961)", "PO (0.937)", "FJ
(0.918)")
with(PCA, legend(x=-2.4,y=2.2, legend = loc, bty = "n", col = "gray32", pch = 21, pt.bg =
colo, cex=1, title = "Site (1-AI)"))

# Add aridity vector
fit <- envfit(pca_all, stem$aridity, permutations = 999)
setas <- fit$vectors$arrows[1, ]
arrows(0, 0, setas[1], setas[2], col = "darkgreen", lwd = 3, length = 0.1)
text(setas[1] * 1.2, setas[2] * 1.2, labels = "1-AI", col = "darkgreen", cex = 1)

dev.off()

#----PERMANOVA ----

com<-vegdist(PCA, method="bray") # También se puede usar "euclidean" si la distancia entre
objetos importa. Se puede usar también adonis() directamente sin crear una matriz de
dissimilitud si los datos están en formato as.matrix()
com<-as.matrix(com)
```

```
# Usando adonis()***
perm<-adonis2(com ~ PCA$Site, method = "bray", data = PCA, permutations = 9999)
perm


# Ternary plot triángulo -----
#-----------------------------

# Data for wood stem/trunck trait data only, including RGR
stem <- read.table("Stem_Traits_lnCWM.txt", header = TRUE)

# Cargar las librerías necesarias
library(Ternary)
library(PlotTools)
library(viridisLite)

names(stem)
anatomic <- stem
str(anatomic)
anat

par(mar = rep(0.3, 4))
TernaryPlot(alab = "Parenchyma (%)", blab = "Lumen (%)", clab = "Fiber (%)", grid.lines =
10)
#  zoom mode, region = list
TernaryPlot(alab = "Parenchyma (%)",
            blab = "Lumen (%)",
            clab = "Fiber (%)",
            grid.lines = 10,
            region = list(min = c(0, 10,50),
                          max = c(50,50,95)))

my_palette <- c("#EE799F", "#EEEE00", "#FF8C00", "#B1E0B7", "#ADDBF4", "#DACEFB")
my_palettelegend <- c("#EE799F", "#FF8C00", "#EEEE00", "#B1E0B7", "#ADDBF4", "#DACEFB")

site_colors <- c(rep("#EE799F", 20), rep("#EEEE00", 20), rep("#FF8C00", 20),
                 rep("#B1E0B7", 20), rep("#ADDBF4", 20), rep("#DACEFB", 20))

# Add points
TernaryPoints(anat, pch = 21, col = "black", bg = site_colors, cex=1.5)

# Legend
One_AI<- c("PA (0.981)", "LLA (0.974)", "QL (0.969)",
           "CHA (0.961)", "PO (0.937)", "FJ (0.918)")
legend("topright",
       legend = One_AI, pch = 21, col = "black", pt.bg = my_palettelegend,
       xjust = 1, yjust = 1, inset = c(0.02, 0.02),
       cex = 1, title = "Site (1-AI)", bty = "n")

range(stem$fibre)
range(stem$parenchyma)
range(stem$total.lumen)


## GLM analyses for Aridity vs. 8 traits
#-----------------------------------------------

# Read CWM-Traits for all sites
stem2<- read.table("trait_data.txt", header = TRUE)
str(stem2)
stem2$aridity<-stem$aridity

# WD ----
WD1 <- glm(WD ~ aridity, data = stem2) #dif sig
anova(WD1, test = "LRT")
summary(WD1)
visreg(WD1)
r2(WD1)
model.sel(WD1)

WD2 <- glm(WD ~ aridity+ I(aridity^2), data = stem2) #dif sig
```

```
anova(WD2, test = "LRT")
summary(WD2)
visreg(WD2)
r2(WD2)
model.sel(WD2)

anova(WD1, WD2, test = "LRT")
model.sel(WD1,WD2)

check_heteroscedasticity(WD1)
check_model(WD1)

# PLOT

site_colors <- c("PA" = "#EE799F", "QL" = "#EEEE00", "LLA" = "#FF8C00",
                 "CHA" = "#B1E0B7", "PO" = "#ADDBF4", "FJ" = "#DACEFB")
legend_colors <- c("PA" = "#EE799F", "LLA" = "#FF8C00", "QL" = "#EEEE00",
                   "CHA" = "#B1E0B7", "PO" = "#ADDBF4", "FJ" = "#DACEFB")

loc <- c("PA", "LLA", "QL", "CHA", "PO", "FJ")
stem$Site <- factor(stem$Site, levels = loc)
point_colors <- site_colors[as.character(stem$Site)]


#---- PLOT WD-----

png("Fig02A.png", width=9, height=8, units="in", res=600)
par(mar = c(5, 6.5, 3, 4), mgp=c(4,1,0))

vis <- visreg(WD2, "aridity", jitter = TRUE, partial = TRUE, overlay = TRUE,
              line = list(col = "red", lwd = 3),
              fill = list(col = "lightgray"),
              points = list(bg = point_colors, col = "black", pch = 21, cex = 1.5),
              xlab = "Aridity (1-AI)",
              ylab = expression("WD (g/cm"^3*")"),
              cex.axis = 1.5, cex.lab = 2)

legend("bottomleft", legend = loc, col = "black",
       pt.bg = legend_colors, pch = 21, pt.cex = 1, cex = 1,
       bty = "n", title = "Sites", ncol = 1, xpd = TRUE, inset = c(0.05, 0.03))
text(0.92, 0.87, "A", cex = 1.5, font = 2)

dev.off()


# Bark ----

corteza1 <- glm(corteza ~ aridity, data = stem2) #dif sig
anova(corteza1, test = "LRT")
summary(corteza1)
visreg(corteza1)
r2(corteza1)
model.sel(corteza1)

corteza2 <- glm(corteza ~ aridity+ I(aridity^2), data = stem2) #dif sig
anova(corteza2, test = "LRT")
summary(corteza2)
visreg(corteza2)
r2(corteza2)
model.sel(corteza2)

anova(corteza1, corteza2, test = "LRT")
model.sel(corteza1,corteza2)

check_heteroscedasticity(corteza1)
check_model(corteza1)

#---- PLOT Bark-----

png("Fig02B.png", width=9, height=8, units="in", res=600)
par(mar = c(5, 6.5, 3, 4), mgp=c(4,1,0))
```

```
vis <- visreg(corteza2, "aridity", jitter = TRUE, partial = TRUE, overlay = TRUE,
              line = list(col = "red", lwd = 3),
              fill = list(col = "lightgray"),
              points = list(bg = point_colors, col = "black", pch = 21, cex = 1.5),
              xlab = "Aridity (1-AI)",
              ylab = "Bark (proportion)",
              cex.axis = 1.5, cex.lab = 2)
text(0.92, 0.235, "B", cex = 1.5, font = 2)
dev.off()


# Fiber ----

fibra1 <- glm(fibra ~ aridity, data = stem2) #dif sig
anova(fibra1, test = "LRT")
summary(fibra1)
visreg(fibra1)
r2(fibra1)
model.sel(fibra1)

fibra2 <- glm(fibra ~ aridity+ I(aridity^2), data = stem2) #dif sig
anova(fibra2, test = "LRT")
summary(fibra2)
visreg(fibra2)
r2(fibra2)
model.sel(fibra2)

anova(fibra1, fibra2, test = "LRT")
model.sel(fibra1,fibra2)
model.avg(fibra1,fibra2)

check_heteroscedasticity(fibra1)
check_model(fibra1)

#---- PLOT Fiber-----

png("Fig02C.png", width=9, height=8, units="in", res=600)
par(mar = c(5, 6.5, 3, 4), mgp=c(4,1,0))

vis <- visreg(fibra2, "aridity", jitter = TRUE, partial = TRUE, overlay = TRUE,
              line = list(col = "red", lwd = 3),
              fill = list(col = "lightgray"),
              points = list(bg = point_colors, col = "black", pch = 21, cex = 1.5),
              xlab = "Aridity (1-AI)",
              ylab = "Fiber (%)",
              cex.axis = 1.5, cex.lab = 2)
text(0.92, 92, "C", cex = 1.5, font = 2)
dev.off()


# Lumen ----

lumen1 <- glm(lumen ~ aridity, data = stem2) #dif sig
anova(lumen1, test = "LRT")
summary(lumen1)
visreg(lumen1)
r2(lumen1)
model.sel(lumen1)

lumen2 <- glm(lumen ~ aridity+ I(aridity^2), data = stem2) #dif sig
anova(lumen2, test = "LRT")
summary(lumen2)
visreg(lumen2)
r2(lumen2)
model.sel(lumen2)

anova(lumen1, lumen2, test = "LRT")
model.sel(lumen1,lumen2)
model.avg(lumen1,lumen2)
```

```
check_heteroscedasticity(lumen1)
check_model(lumen1)

#---- PLOT Lumen-----

png("Fig02D.png", width=9, height=8, units="in", res=600)
par(mar = c(5, 6.5, 3, 4), mgp=c(4,1,0))

vis <- visreg(lumen2, "aridity", jitter = TRUE, partial = TRUE, overlay = TRUE,
              line = list(col = "red", lwd = 3),
              fill = list(col = "lightgray"),
              points = list(bg = point_colors, col = "black", pch = 21, cex = 1.5),
              xlab = "Aridity (1-AI)",
              ylab = "Lumen (%)",
              cex.axis = 1.5, cex.lab = 2)
text(0.92, 39, "D", cex = 1.5, font = 2)
dev.off()


# Parenchyma ----

parenquima1 <- glm(parenquima ~ aridity, data = stem2) #dif sig
anova(parenquima1, test = "LRT")
summary(parenquima1)
visreg(parenquima1)
r2(parenquima1)
model.sel(parenquima1)

parenquima2 <- glm(parenquima ~ aridity+ I(aridity^2), data = stem2) #dif sig
anova(parenquima2, test = "LRT")
summary(parenquima2)
visreg(parenquima2)
r2(parenquima2)
model.sel(parenquima2)

anova(parenquima1, parenquima2, test = "LRT")
model.sel(parenquima1,parenquima2)

check_heteroscedasticity(parenquima1)
check_model(parenquima1)


#---- PLOT Parenchyma-----

png("Fig02E.png", width=9, height=8, units="in", res=600)
par(mar = c(5, 6.5, 3, 4), mgp=c(4,1,0))

vis <- visreg(parenquima2, "aridity", jitter = TRUE, partial = TRUE, overlay = TRUE,
              line = list(col = "red", lwd = 3),
              fill = list(col = "lightgray"),
              points = list(bg = point_colors, col = "black", pch = 21, cex = 1.5),
              xlab = "Aridity (1-AI)",
              ylab = "Parenchyma (%)",
              cex.axis = 1.5, cex.lab = 2)
text(0.92, 13.55, "E", cex = 1.5, font = 2)
dev.off()


# Vessels ----

vasos1 <- glm(vasos ~ aridity, data = stem2) #dif sig
anova(vasos1, test = "LRT")
summary(vasos1)
visreg(vasos1)
r2(vasos1)
model.sel(vasos1)

vasos2 <- glm(vasos ~ aridity+ I(aridity^2), data = stem2) #dif sig
anova(vasos2, test = "LRT")
summary(vasos2)
visreg(vasos2)
```

```
r2(vasos2)
model.sel(vasos2)

anova(vasos1, vasos2, test = "LRT")
model.sel(vasos1,vasos2)
model.avg(vasos1,vasos2)

check_heteroscedasticity(vasos1)
check_model(vasos1)

#---- PLOT non-vessels-----

png("Fig02F.png", width=9, height=8, units="in", res=600)
par(mar = c(5, 6.5, 3, 4), mgp=c(4,1,0))

vis <- visreg(vasos2, "aridity", jitter = TRUE, partial = TRUE, overlay = TRUE,
              line = list(col = "red", lwd = 3),
              fill = list(col = "lightgray"),
              points = list(bg = point_colors, col = "black", pch = 21, cex = 1.5),
              xlab = "Aridity (1-AI)",
              ylab = "non-vessels (%)",
              cex.axis = 1.5, cex.lab = 2)
text(0.92, 10, "F", cex = 1.5, font = 2)
dev.off()

# MVD ----

MVD1 <- glm(MVD ~ aridity, data = stem2) #dif sig
anova(MVD1, test = "LRT")
summary(MVD1)
visreg(MVD1)
r2(MVD1)
model.sel(MVD1)

MVD2 <- glm(MVD ~ aridity+ I(aridity^2), data = stem2) #dif sig
anova(MVD2, test = "LRT")
summary(MVD2)
visreg(MVD2)
r2(MVD2)
model.sel(MVD2)

anova(MVD1, MVD2, test = "LRT")
model.sel(MVD1, MVD2)


#---- PLOT MVD-----

png("Fig02G.png", width=9, height=8, units="in", res=600)
par(mar = c(5, 6.5, 3, 4), mgp=c(4,1,0))

vis <- visreg(MVD2, "aridity", jitter = TRUE, partial = TRUE, overlay = TRUE,
              line = list(col = "red", lwd = 3),
              fill = list(col = "lightgray"),
              points = list(bg = point_colors, col = "black", pch = 21, cex = 1.5),
              xlab = "Aridity (1-AI)",
              ylab = expression("MVD ("*mu*"m)"),
              cex.axis = 1.5, cex.lab = 2)
text(0.92, 34, "G", cex = 1.5, font = 2)
dev.off()


# VD ----

VD1 <- glm(DV ~ aridity, data = stem2) #dif sig
anova(VD1, test = "LRT")
summary(VD1)
visreg(VD1)
r2(VD1)
model.sel(VD1)

VD2 <- glm(DV ~ aridity+ I(aridity^2), data = stem2) #dif sig
```

```
anova(VD2, test = "LRT")
summary(VD2)
visreg(VD2)
r2(VD2)
model.sel(VD2)

anova(VD1, VD2, test = "LRT")
model.sel(VD1, VD2)

check_heteroscedasticity(VD1)
check_model(VD1)

#---- PLOT VD-----

png("Fig02H.png", width=9, height=8, units="in", res=600)
par(mar = c(5, 6.5, 3, 4), mgp=c(4,1,0))

vis <- visreg(VD2, "aridity", jitter = TRUE, partial = TRUE, overlay = TRUE,
              line = list(col = "red", lwd = 3),
              fill = list(col = "lightgray"),
              points = list(bg = point_colors, col = "black", pch = 21, cex = 1.5),
              xlab = "Aridity (1-AI)",
              ylab = expression("VD (vessels/"*mu*"m"^2*")"),
              cex.axis = 1.5, cex.lab = 2)
text(0.92, 0.52, "H", cex = 1.5, font = 2)
dev.off()


# Functional spectra vs. Aridity
#--------------------------------

#--- PC1 vs. Aridity ----

PC1_l <- glm(PC1 ~ aridity, data = stem2) #dif sig
anova(PC1_l, test = "LRT")
summary(PC1_l)
visreg(PC1_l)
r2(PC1_l)
model.sel(PC1_l)

PC1_q <- glm(PC1 ~ aridity+ I(aridity^2), data = stem2) #dif sig
anova(PC1_q, test = "LRT")
summary(PC1_q)
visreg(PC1_q)
r2(PC1_q)
model.sel(PC1_q)

anova(PC1_l, PC1_q, test = "LRT")
model.sel(PC1_l, PC1_q)
model.avg(PC1_l, PC1_q)

# Plot PC1

par(mar = c(5, 5, 4, 5) + 0.1, mgp=c(3,1,0), cex.lab = 1.5)
vis <- visreg(PC1_q, "aridity", jitter = TRUE, partial = TRUE,
              overlay = TRUE, scale="response",
              line = list(col = "red", lwd = 3),
              fill = list(col = "lightgray"),
              points = list(bg = point_colors, col = "black", pch = 21, cex = 1.5),
              xlab = "Aridity (1-AI)",
              ylab = "PC1 - Resource allocation gradient")


#--- PC2 vs. Aridity ----

PC2_l <- glm(PC2 ~ aridity, data = stem2) #dif sig
anova(PC2_l, test = "LRT")
summary(PC2_l)
visreg(PC2_l)
r2(PC2_l)
model.sel(PC2_l)
```

```
PC2_q <- glm(PC2 ~ aridity+ I(aridity^2), data = stem2) #dif sig
anova(PC2_q, test = "LRT")
summary(PC2_q)
visreg(PC2_q)
r2(PC2_q)
model.sel(PC2_q)

anova(PC2_l, PC2_q, test = "LRT")
model.sel(PC2_l, PC2_q)

#--- Plot PC2 -----

par(mar = c(5, 5, 4, 5) + 0.1, mgp=c(3,1,0), cex.lab = 1.5)
vis <- visreg(PC2_q, "aridity", jitter = TRUE, partial = TRUE,
              overlay = TRUE, scale="response",
              line = list(col = "red", lwd = 3),
              fill = list(col = "lightgray"),
              points = list(bg = point_colors, col = "black", pch = 21, cex = 1.5),
              xlab = "Aridity (1-AI)",
              ylab = "PC2 - Hydraulic vulnerability / efficiency gradient")

legend("bottomleft", legend = loc, col = "black",
       pt.bg = legend_colors, pch = 21, pt.cex = 1, cex = 1,
       bty = "n", title = "Sites", ncol = 1, xpd = TRUE,
       inset = c(0.05, 0.03))


# WD and fractions multiple regression-----
#------------------------------------------

WDi <- glm(WD ~ fibra + parenquima + lumen, data = stem2) #dif sig
global<-dredge(WDi)
anova(WDi, test = "Chisq")
summary(WDi)
r2(WDi)
model.avg(global)

check_heteroscedasticity(WDi)
check_model(WDi)

#---Plot

par(mar = c(5, 5, 4, 5) + 0.1, mgp=c(3,1,0), cex.lab = 1.5)
vis <- visreg(WDi, "fibra", jitter = TRUE, partial = TRUE,
              overlay = TRUE, scale="response",
              line = list(col = "red", lwd = 3),
              fill = list(col = "lightgray"),
              points = list(bg = point_colors, col = "black", pch = 21, cex = 1.5),
              xlab = expression("WD (g/cm"^3*")"),
              ylab = "Fibre")

legend("bottomleft", legend = loc, col = "black",
       pt.bg = legend_colors, pch = 21, pt.cex = 1, cex = 1,
       bty = "n", title = "Sites", ncol = 1, xpd = TRUE,
       inset = c(0.05, 0.03))

# 2D graph-----

png("Fig03b.png", width=9, height=8, units="in", res=600)
visreg2d(WDi,
         x = "fibra",
         y = "parenquima",
         plot.type = "gg",
         xlab = "Fiber (%)",
         ylab = "Parenchyma (%)",
         zlab = expression("WD (g/cm"^3*")"),
         cex.lab = 2,
         color = RColorBrewer::brewer.pal(3, "OrRd"), alpha.f = 0.2,
         scale = "response") +
  geom_point(data = stem2, # Specify the data frame for points
```

```
             aes(x = fibra, y = parenquima, z = WD), # Corrected aesthetics
             shape = 21,   # Filled circle (allows separate fill/color control)
             colour = "black",       # Outline color
             fill = point_colors,   # Inner fill color
             size = 4,              # Double the default size (default is ~1)
             stroke = 0.8,          # Outline thickness
             inherit.aes = FALSE) +
  scale_x_reverse() +  # This inverts the x-axis
  theme_minimal() + # Optional: add a clean theme
  theme(
    axis.title.x = element_text(size = 16),  # Double default size (typically 8)
    axis.title.y = element_text(size = 16),
    axis.text.x = element_text(size = 14),   # Also increase tick labels
    axis.text.y = element_text(size = 14)
  )
dev.off()
```
